# Assessment of Nonverbal and Verbal Apraxia in Patients with Parkinson's Disease

**DOI:** 10.1155/2015/840327

**Published:** 2015-10-12

**Authors:** Monia Presotto, Maira Rozenfeld Olchik, Artur Francisco Shumacher Shuh, Carlos R. M. Rieder

**Affiliations:** ^1^Graduate School of Medicine: Medical Sciences, Federal University of Rio Grande do Sul (UFRGS), Brazil; ^2^Department of Speech Pathology, UFRGS, Brazil; ^3^Movement Disorders Group, The Clinical Hospital of Porto Alegre (HCPA), Ramiro Barcelos 2350, 90035-903 Porto Alegre, RS, Brazil; ^4^The Movement Disorders Clinic, The Clinical Hospital of Porto Alegre (HCPA), Ramiro Barcelos 2350, 90035-903 Porto Alegre, RS, Brazil; ^5^The Federal University of Health Sciences of Porto Alegre (UFCSPA), Brazil

## Abstract

*Objective*. To assess the presence of nonverbal and verbal apraxia in patients with Parkinson's disease (PD) and analyze the correlation between these conditions and patient age, education, duration of disease, and PD stage, as well as evaluate the correlation between the two types of apraxia and the frequency and types of verbal apraxic errors made by patients in the sample. *Method*. This was an observational prevalence study. The sample comprised 45 patients with PD seen at the Movement Disorders Clinic of the Clinical Hospital of Porto Alegre, Brazil. Patients were evaluated using the Speech Apraxia Assessment Protocol and PD stages were classified according to the Hoehn and Yahr scale. *Results*. The rate of nonverbal apraxia and verbal apraxia in the present sample was 24.4%. Verbal apraxia was significantly correlated with education (*p* ≤ 0.05). The most frequent types of verbal apraxic errors were omissions (70.8%). The analysis of manner and place of articulation showed that most errors occurred during the production of trill (57.7%) and dentoalveolar (92%) phonemes, consecutively. *Conclusion*. Patients with PD presented nonverbal and verbal apraxia and made several verbal apraxic errors. Verbal apraxia was correlated with education levels.

## 1. Introduction

Parkinson's disease (PD) is a chronic neurological condition of idiopathic nature, whose typical symptoms are rigidity, bradykinesia, resting tremor, and postural instability. There are poor control of voluntary movement, dyskinesias, autonomic abnormalities, and superimposed age-related changes in neuromuscular and musculoskeletal systems [1, 2].

The speech pathology literature has also described voice and speech abnormalities in PD [3–7]. According to Vitorino and Homem [8], speech is among the most severely affected abilities in individuals with PD. These impairments are characterized by slow, weak, imprecise, or uncoordinated movements of the speech musculature caused by alterations in the motor control or planning of speech articulation, which involves brain structures such as the basal ganglia, the cerebellum, the supplementary motor area, and frontal circuitries. The combination of rigidity and bradykinesia, associated with impairments in sensorial and cognitive processing, triggers changes in speech (dysphonia and dysarthria) at some stage of the disease. These modifications along lead to a particular kind of condition called hypokinetic dysarthria [9–11]. Voice disorders have a significant functional impact on patients with PD [12].

Dysarthria is the most common acquired speech disorder in PD, and the most extensively studied in this population. However, some studies have also identified the presence of apraxia of speech in patients with PD [13, 14].

Nonverbal apraxia, also known as orofacial or buccofacial apraxia, is characterized by impairments in sequencing voluntary nonverbal movements of the tongue, lips, jaw, and other associated orofacial structures, while verbal apraxia, or apraxia of speech, involves impairments in the sequencing of articulatory movements required for speech production [15].

Although verbal apraxia is most commonly associated with impairments in articulation and prosody, it may also involve dysfunctions in other aspects of speech production: respiration, resonance, and phonation [16, 17]. Patients with apraxia typically display inconsistent errors during voluntary speech, with anticipative errors being more common than perseverative ones [18].

Nonverbal apraxia and verbal apraxia have only been sparsely studied in patients with PD. To the authors' knowledge, no studies have focused on the verbal praxic errors made by patients with PD or classified these errors according to the manner and place of articulation. Therefore, the goal of this study was to assess the presence of nonverbal and verbal apraxia in patients with PD. The presence of nonverbal and verbal apraxia and their correlation with age, education, duration of disease, and PD stage (Hoehn and Yahr scale) were also investigated, as were the correlation between nonverbal and verbal apraxia and the different types of praxic errors made by patients in the sample.

## 2. Method

### 2.1. Patients and Method

This was a quantitative, observational, and descriptive study involving 45 patients with idiopathic PD seen at the Movement Disorders Clinic of the Clinical Hospital of Porto Alegre (HCPA), RS, Brazil, between April 2012 and November 2013. All patients signed an informed consent form prior to participation. The present study was approved by the HCPA Research Ethics Committee under project number 100501.

The sample consisted of patients with a medical diagnosis of idiopathic PD who were receiving pharmacological treatment for their condition at the aforementioned clinic. Patients with oral comprehension difficulties, with hearing or visual impairments which interfered with the completion of assessment tasks, who were off medication at the time of study, who had previously had a speech therapy, surgery, and comorbidities, or who did not agree to participate were excluded from the sample.

Data was collected through a clinical interview, a PD classification instrument [19], and the Speech Apraxia Assessment Protocol [20]. The following data were collected in the interview: patient name, gender, date of birth, age, education, occupation, time since PD diagnosis, medication used, and medication status at the time of assessment.

PD was classified using the Hoehn and Yahr (H&Y) Degree of Disability Scale, which was developed in 1967 and provides a quick and practical assessment of patient status. The scale is divided into five categories of severity. Stages 1, 2, and 3 correspond to mild to moderate incapacitation, while stages 4 and 5 are indicative of severe incapacitation [19].

Nonverbal apraxia and verbal apraxia were evaluated using the Speech Apraxia Assessment Protocol [20], the only published instrument which allows for the diagnosis of these conditions in Brazilian Portuguese-speaking patients. This protocol was compiled and adapted from several instruments used to assess apraxia and evaluates several symptoms of nonverbal apraxia and verbal apraxia, all of which were considered in the present study. Since most of the instruments used to evaluate apraxia were originally published in English, their wording was both translated and adapted to Brazilian Portuguese during the construction of the protocol, since translation alone may not have been sufficient to ensure that the resulting instrument would apply to the language and culture of Brazilian patients with apraxia. This protocol can be considered a sensitive diagnostic measure of apraxia of speech following brain damage.

Nonverbal apraxia was evaluated based on the performance of the following 20 movements: showing teeth, smiling, protruding the tongue, blowing, raising tongue, biting lower lip, clearing throat, lowering the tongue, protruding/retracting the tongue, puckering lips, coughing, puffing out cheeks, moving the tongue laterally in the mouth, raising and lowering the tongue, blowing a kiss, alternating between puckering lips and smiling, moving the jaw from side to side, running the tongue over one's lips, clicking the tongue, and moving the tongue sideways and up. Instructions were given orally and supplemented with articulatory cues if patients were unable to perform the movements in response to verbal commands alone. Performance on each item was scored on a categorical scale. The maximum obtainable score on the task is 200. Scores between 160 and 200 were considered normal, while those between 80 and 159 were considered indicative of mild nonverbal apraxia, scores 40 and 79 suggested moderate nonverbal apraxia, and scores of 39 or less suggested severe nonverbal apraxia. Verbal abilities were assessed through the following tasks: word and sentence repetition, spontaneous speech production, automatic speech, and reading aloud of words and sentences. In the repetition task, patients were read a series of words and sentences and asked to repeat them aloud. Spontaneous speech production was evaluated by giving participants at least two minutes to describe the scene depicted on a picture card. Automatic speech was assessed by asking patients to count from 1 to 20 and name the months of the year. In the reading task, participants were asked to read a series of words and sentences out loud. Performance was evaluated quantitatively and qualitatively based on the errors made on each of the tasks. The number of verbal errors was summed to provide a quantitative measure of performance.

Verbal praxic errors were then classified into the following four types: addition (A), substitution (S), repetition (R), and omission (O). A-errors were defined as the addition of a phoneme or syllable to a specific word. S-errors consisted of single phoneme substitutions, while R-errors occurred when participants repeated a sound, full word or part of a word, or a section of the task instructions more than once. Lastly, O-errors were defined as the omission of a phoneme or syllable. Sequencing errors were also analyzed and classified. Anticipatory errors consist of the anticipation of phonemes in a word; reiteration refers to the repetition of phonemes within the same word, while metathesis is the sequential inversion of phonemes in a word.

Verbal apraxia was also assessed based on the manner and place of articulation of different phonemes, based on the International Phonetic Alphabet (IPA-2005). The nature of the airflow through supraglottic cavities (pharynx, oral, and nasal cavities) during the production of a phoneme defines its manner of articulation (nasal, occlusive, fricative, lateral, or trill). Consonants can also be characterized in terms of their place of articulation, or the location at which the airflow is obstructed or constricted during phoneme production. This allows for the classification of consonants as bilabial, labiodental, dentoalveolar, alveolopalatal, velar, uvular, and glottal.

It is important to note that although the initial aim of the present study was to evaluate nonverbal apraxia and verbal apraxia, we also investigated any alterations in the five main parameters of speech (respiration, phonation, resonance, articulation, and prosody), which could characterize dysarthria. These symptoms were evaluated using an adapted form of the Central Dysarthria Assessment Protocol for Patients with Parkinson's disease, which was adapted for use in Brazilian Portuguese by Fracassi et al. [21]. This instrument assesses respiration based on the presence of pneumophonoarticulatory incoordination, phonation in terms of voice quality, onset, intensity, and pitch, and resonance in terms of hypernasality, in addition to alterations in articulation and prosody. Three blind evaluators (specialist judges in the field of Speech-Language Pathology) analyzed recorded videos of the patients and, consensually, concluded if they were apraxic and then either dysarthric or not.

### 2.2. Sample Size Calculation

To detect apraxia, whose prevalence was set at 38.9% according to Howard et al. [14], with a significance of 5% and an error rate of 15%, a sample of 41 patients was required.

### 2.3. Statistical Analysis

Results were analyzed by simple and cross-tabulation, graphs, and descriptive statistics. Spearman coefficients were used to evaluate the correlation between quantitative variables (age, education, and duration of PD), nonverbal and verbal apraxia, and between the two types of apraxia. Gender and PD stage (H&Y) were compared between patients with verbal and nonverbal apraxia using the Mann-Whitney Test. Results were considered significant at 5% (*p* ≤ 0.05). Data were analyzed using the SPSS (Statistical Package for the Social Science) software, version 18.0.

## 3. Results

### 3.1. General and Clinical Characteristics of Patients with PD

The sample consisted of 45 patients with PD (62.2% male). The prevalence of nonverbal apraxia and verbal apraxia in the present sample was 24.4% (11 patients). The mean and minimum and maximum values for age, education, duration of disease, and PD stage (H&Y) are shown in Table 1.

### 3.2. Characteristics of Patients without and with Nonverbal and Verbal Apraxia

The mean and minimum and maximum values of age, education, duration of disease, stage of PD (H&Y), and gender are shown in Table 2, as are the results of comparisons between patients with and without apraxia.

### 3.3. Nonverbal Apraxia

Participants were classified according to the severity of nonverbal apraxia using a specialized assessment protocol [20]. All participants (100%) with the condition presented with a mild form of the disorder.

These individuals had the most difficulty with the following movements: jaw lateralization, smiling, tongue lateralization, and moving the tongue up and down. All participants with apraxia had difficulty performing both simple and sequential movements.

### 3.4. Verbal Apraxia

Table 3 shows the frequency of verbal praxic errors identified in the sample.

The most commonly identified errors in the manner of articulation were noted in the production of trill (57.7%), followed by fricative (19.2%), occlusive (9.6%), lateral (9.6%), and nasal (3.9%) phonemes. The classification of errors according to the place of articulation revealed that most errors were identified in dentoalveolar consonants (92.2%), followed by velar (3.1%), bilabial (3.1%), and alveolopalatal (1.6%) ones. Although one sequential error was observed, no reiteration or metathesis errors were noted in the sample.

Therefore, patients missed words, sentences, and spontaneous speech repetitions, although none of the 45 patients had any difficulty in automatic speech tasks, such as counting from 1 to 20 and saying the months of the year. Verbal apraxia was diagnosed in 100% (11 patients) of patients with nonverbal apraxia.

### 3.5. Comparison and Correlation between Nonverbal Apraxia and Verbal Apraxia

The comparison between the PD stages (H&Y) and genders of patients with nonverbal apraxia versus verbal apraxia is shown in Table 4.

Nonverbal apraxia was not significantly correlated with patient age, education, or the duration of the disease. Although verbal apraxia was significantly correlated with patient education, this was not the case for patient age or the duration of disease, as shown in Table 5.

Verbal apraxia was present in 100% of patients (11 patients) with nonverbal apraxia. However, the correlation between nonverbal apraxia and verbal apraxia was nonsignificant (*r*_*s*_ = 0.318, *p* = 0.340).

## 4. Discussion

In this study, the presence of verbal and nonverbal apraxia in patients with PD was detected. Individuals with nonverbal apraxia were found to have a mild form of the condition. All patients with PD sorted as apraxic were also sorted as dysarthric and made several praxic errors during speech production.

The present findings corroborated those of Howard et al. [14], who found that speech impairments in PD are not limited to dysarthria, which results from alterations in muscle control, but may also involve apraxia, which is caused by alterations in the planning and programming of speech. Of the 36 patients evaluated, 23 were diagnosed with dysarthria, while 14 had apraxia of speech. All patients with apraxia also had dysarthria. Apraxia was only present in a subgroup of patients who also presented with dysarthria, suggesting that the former condition may only occur in the presence of the latter in patients with PD. These findings illustrate the importance of investigating these individuals for apraxia of speech.

Although the assessment protocols used in the present study focused primarily on the evaluation of nonverbal apraxia and verbal apraxia, symptoms of dysarthria were also investigated. Mac-Kay [22] defined dysarthria as a speech disorder caused by neurological impairment and is characterized by weakness, slowness, or incoordination of the speech musculature. In dysarthria, speech programming is intact, but motor execution is impaired. Dysarthria usually affects the five major aspects of speech production (respiration, phonation, resonance, articulation, and prosody).

It is noteworthy to point out that all the patients in the present study were in the on phase of their medication during assessment. However, a number of studies in the literature have addressed the issues of use of medications for movement improvement in PD and the fluency sequelae induced, although no consensus has been reached among studies on motor production of speech, with some showing improvements, and others deterioration in the on phase [23–25].

Investigators who evaluated 45 subjects with idiopathic PD found no evidence of nonverbal (orofacial) apraxia in any patients in the sample. The difference between these findings and those of the present study may be attributable to the fact that participants in the present sample were older (64.9 years versus 62.6 years) and had a longer disease duration (8.3 versus 5.4 years) than those evaluated by Leiguarda et al. [26].

All patients with nonverbal apraxia had difficulty performing both simple gestures and movement sequences. Most of the errors made by these individuals were observed during jaw lateralization, smiling, tongue lateralization, and moving the tongue up and down. The errors observed in tasks assessing nonverbal apraxia may have been caused by alterations in the programming and sequencing of movements, by the motor impairment associated with PD, or have been influenced by both factors. In a previous study, investigators assessed orofacial apraxia in 44 patients with Parkinsonian syndromes, of whom 12 had idiopathic Parkinson's disease (IPD), 8 presented with multiple systems atrophy (MSA), 12 had supranuclear progressive paralysis (SPP), and 12 had been diagnosed with corticobasal degeneration (CBD). Patients with CBD were significantly more impaired than those with IPD, MSA, or SPP. The patients in this study showed significant impairment in the performance of gesture sequences [27]. No studies in the literature have described the specific movements associated with the greatest levels of impairment.

Articulation errors and prosody alterations are common features of verbal apraxia. Alterations in prosody were identified in all apraxic patients in the present study, as were articulation errors. According to the literature, the most common speech errors observed in patients with apraxia are substitutions, additions, repetitions, and phonemic elongation, all of which are produced in a highly irregular pattern [9, 28]. The present findings were largely consistent with these observations, save for the frequency of omissions, which were the most common errors in the present sample, followed by substitutions, repetitions, and additions. More omission mistakes were found, because PD patients showed slow, weak, and uncoordinated movements of the muscles required to speak, with the concatenated output of phonetic segments becoming harder [11].

According to Wertz et al. [29], patients with verbal apraxia may present excessive effort for voice emission, self-corrections, prosody alterations, inconsistent articulation errors when repeating the same stimulus more than once, and difficulty initiating sentences. These observations are in agreement with the present findings. Our sample of patients with apraxia showed self-correction, hesitation, and prosody alterations throughout the study.

Cera and Ortiz [30] performed a phonological analysis of substitution and omission errors in a sample of 20 Brazilian Portuguese-speaking adults with apraxia of speech following single left hemisphere lesions, using an apraxia assessment protocol. The authors identified consonant, but not vowel, production errors. Most errors in the manner of articulation were observed in the production of liquid/lateral phonemes, followed by fricatives, occlusives, and trills. No errors were observed in nasal phonemes. The classification of errors by place of articulation revealed that most errors were made in the production of dentoalveolar phonemes, followed by velar, bilabial, and labiodental ones.

Although the aforementioned study did not focus exclusively on patients with PD, its findings are in agreement with those of the present study, in which only consonant errors were identified. The higher frequency of consonant errors as compared to vowel errors has already been described in the literature [15, 30–32]. This may be explained by the fact that vowels are more frequently used in speech and more easily produced than consonants, since they only require a simple adjustment of articulators and the performance of slow articulator movements [18]. The classification of errors according to the manner of articulation revealed somewhat different results. Trills phonemes displayed the most errors, followed by fricative, occlusive, liquid/lateral, and nasal ones. Similar results were obtained following the classification of errors by place of articulation. Dentoalveolar phonemes appeared to yield the most errors, followed by velar, bilabial, and alveolopalatal ones. The patients with PD showed more mistakes in the trills due to the presence of the bradykinesia and rigidity which caused a decrease in the mobility and a movement directions, resulting in the loss of ability to produce the phonemes correctly [33].

No studies in the literature have evaluated the correlation between nonverbal apraxia and verbal apraxia in patients with PD and variables such as age, education, disease duration, and stage of the disease. However, Brabo et al. [34] evaluated the frequency and types of disfluency displayed by 30 patients with IPD and 30 healthy adults, in addition to participant age, gender, education, duration of disease, Hoehn and Yahr scores, and cognitive status. The number of total and atypical disfluencies was higher among patients with PD, and the groups did not differ with regard to age, education, and gender. However, disease duration and age were both associated with the occurrence of disfluencies. In the present study, nonverbal apraxia was not significantly correlated with age, education, or duration of PD. Additionally, patients with nonverbal and verbal apraxia did not differ with regard to gender or PD stages (H&Y). Although verbal apraxia was significantly associated with education levels, it did not correlate with age or disease duration. The outcomes of this study differ because aging is related with the increase of hesitations, interjections, and pauses in the speech, and as the disease evolves and the clinical situation gets worse, a predominance of unusual disfluencies occurs in the speech of the PD patients [35].

The negative association between verbal apraxia and education may reflect an influence of educational level with the verbal apraxia. Another hypothesis would be the impact of education on the course of progressive disease. The influence of education on brain functioning has been suggested. Education may be a fundamental contributor to cognitive reserve [36]. Studies have also shown that higher levels of education may delay the clinical manifestation and attenuate the severity of conditions such as dementia. Therefore, education would have a protective effect against neurological disorders. The association between younger age and/or more years of education and better performance on measures of attention, memory, and reasoning is already well established in the literature. The cognitive stimulation is something possible due to the brain plasticity, affecting the functional network organization in a good way [37, 38]. Therefore, in light of these data and the present results, we hypothesize that patients with PD who have lower education levels may be more likely to develop verbal apraxia.

In clinical practice, many patients who present with nonverbal apraxia also have verbal apraxia [15]. These conditions are comorbid in approximately 48% of cases [39]. The association between the two disorders was far stronger in the present study, since 100% of patients with nonverbal apraxia also displayed verbal praxic errors. However, there was no direct correlation between nonverbal apraxia and verbal apraxia (*p* = 0.340). This association may be attributable to the fact that the sample was entirely composed of patients with PD, who may have particular difficulty articulating speech sounds.

In light of the presence of nonverbal and verbal apraxia in patients with PD in the present study, we now present a brief discussion of the association between these conditions.

Apraxia can be caused by lesions in several cortical and subcortical areas. According to the literature, the apraxic symptoms traditionally observed following left or right post-Rolandic damage may also be caused by white matter or subcortical lesions (more specifically, by thalamic and basal nuclei lesions). The physiopathology of apraxia is multifactorial and involves variables as diverse as the functional balance between cerebral hemispheres, sensory processing, and selective attention. The role of intra- and interhemispheric disconnection and neurochemical imbalance in the development of apraxia must also be considered [40].

The apraxia may develop in patients with PD due to the effects of the disease on the basal ganglia, as a result of additional cerebral cortical pathology, or as a consequence of both these factors [26]. As such, apraxia in patients with PD cannot always be attributable to basal ganglia dysfunction alone. Ideomotor apraxia and, less frequently, nonverbal and ideational apraxia are often observed when basal ganglia damage intrude into the adjacent white matter to involve association fibers, especially in the superior longitudinal fasciculus and frontostriatal connections [13, 41]. White matter appears to play a fundamental role in the development of apraxia in patients with deep brain lesions. Cortical dysfunction in PD may reflect damage to ascending noradrenergic, serotonergic, and cholinergic fibers. Damage to the dopaminergic system and the cerebral cortex has also been associated with PD. Amyloid plaques and neurofibrillary tangles, which are typically seen in Alzheimer's disease, as well as Lewy bodies have also been found in the cerebral cortex of patients with PD, regardless of the presence of dementia [42–44].

The apraxia of speech may be associated with damage to areas such as the precentral gyrus and the opercular region of the left hemisphere [45]. The neural basis of voluntary movement disorders has not been entirely elucidated, and apraxia continues to present a challenge to both neurobiological research and clinical practice [46]. The prefrontal cortex (premotor and supplementary motor areas) and its subcortical projections have been especially implicated in apraxia of speech, and some studies suggest that the loss of gray matter in the left medial frontal gyrus, that is, Brodmann's area 46, may be particularly associated with this condition [47]. Apraxia is generally caused by damage to the postcentral parietal lobe which may extend to temporal regions [48].

The nonverbal apraxia is more likely and severe following cortical lesions than basal ganglia damage [13]. However, it may also be caused by alterations in neural networks affecting both cortical and subcortical structures [49]. Nonverbal apraxia is predominantly observed following anterior cortical lesions, although posterior (temporal and parietal) lesions have also been found to affect the performance of sequential movements [50, 51].

PD is caused by a reduction in dopaminergic neurotransmission in the basal ganglia accompanied by the degeneration of pigmented neurons, especially those in the substantia nigra, which results in widespread damage to cerebral functioning. Hence, other nondopaminergic aspects have been involved with the PD as well. Studies with apes have shown explanatory models to the physiopathology of the disease detailing the interrelationships of the dopaminergic direct and indirect aspects. The hypokinetic properties of PD would be the result of the increase of inhibitory exits of the intern globus pallidus, causing the increase in the excitatory exits of the subthalamic nucleus [52]. The findings in the literature and the results of the present study suggest that nonverbal apraxia and verbal apraxia are important disorders, which can occur following cortical lesions, subcortical damage, or both. These conditions can therefore be present in PD and must be further explored in this population [53, 54].

The present study had some limitations. The fact that all patients presented with mild apraxia may be attributable to the fact that the sample was entirely composed of patients with stages 2 (no balance alterations) and 3 PD (with balance alteration). Had patients with stage 4 or 5 PD been evaluated, we hypothesize that more severe apraxia could be identified.

The present study is one of the few in the literature to evaluate both nonverbal apraxia and verbal apraxia in patients with PD. At the time of writing, no additional studies have evaluated the verbal praxic errors made by patients with PD or classified these errors according to the manner and place of articulation. The description of apraxia of speech symptoms provided by the present study makes a significant contribution to the accuracy of phonoaudiological assessment and has important implications for clinical practice and future research. The present study also raised several questions regarding the assessment of speech articulation in patients with neurodegenerative diseases and pointed to the need of specific assessment protocols for these populations, since no such instruments have been found in the literature. Further research might also happen such as to repeat the study with more severe patients H&Y 4, 5 and an on/off medication group. More detailed assessments of speech alterations may contribute both to a more precise diagnosis and to more adequate therapeutic planning, leading to longer-lasting improvements in patient communicative ability and quality of life.

## 5. Conclusion

The present findings showed a high prevalence of nonverbal apraxia and verbal apraxia in patients with PD and a significant correlation between verbal apraxia and education. Patients with PD made several verbal praxic errors, the most common of which were omissions. The classification of errors by manner and place of articulation revealed that the production of trill and dentoalveolar phonemes, respectively, was the most challenging for participants in the present sample.

## Figures and Tables

**Table 1:** Demographic and clinical characteristics of the sample.

		*N*		Values	Min	Max
		Valid	Missing			
Age		45	0	64.9 (11.3)	35	87

Education		45	0	7.5 (4.6)	0	16

Duration of PD		45	0	8.3 (6.0)	1	25

H&Y	2	24	0	53.3%	—	—
	3	21	0	46.7%	—	—

*N*: sample size; values: mean and standard deviation or percentage; Min: minimum; Max: maximum.

**Table 2:** Assessment of age, education, duration of disease, PD stage (H&Y), and gender in patients without apraxia or with nonverbal and verbal apraxia.

		Classification	*N*		Values	Min	Max
			Valid	Missing			
Age		No apraxia	34	0	64.4 (11.1)	35	87
		Apraxia	11	0	66.3 (12.2)	51	85

Education		No apraxia	34	0	8.1 (4.4)	1	16
		Apraxia	11	0	5.4 (4.7)	0	14

Duration of PD		No apraxia	34	0	7 (4.3)	1	18
		Apraxia	11	0	12.3 (8.7)	1	25

H&Y	2	No apraxia	21	0	61.8%	—	—
		Apraxia	3	0	27.3%		
	3	No apraxia	13	0	38.2%	—	—
		Apraxia	8	0	72.7%		

Gender	M	No apraxia	20	0	58.8%	—	—
		Apraxia	8	0	72.7%		
	F	No apraxia	14	0	41.2%	—	—
		Apraxia	3	0	27.3%		

*N*: sample size; values: mean and standard deviation or percentage; Min: minimum; Max: maximum; M: male; F: female.

**Table 3:** Types of verbal apraxic errors.

Type of error	Total	Words	Sentences	Spontaneous speech	Reading
O	70.8%	56.5%	8.7%	21.7%	13.1%
S	16.9%	100%	0%	0%	0%
R	9.2%	0%	33.3%	50%	16.7%
A	3.1%	100%	0%	0%	0%

O: omission; S: substitution; R: repetition; A: addition.

**Table 4:** Comparison of PD stages (H&Y) and gender between patients with verbal and nonverbal apraxia.

		Total apraxia			
		Nonverbal apraxia		Verbal apraxia	
H&Y	2	155 [150; 158]	(*p* = 0.124)	4 [3; 21]	(*p* = 0.084)
	3	143 [137.5; 150.75]		16.5 [5.25; 27.75]	

Gender	M	146.5 [138.5; 150.75]	(*p* = 0.219)	19.5 [7.5; 27.75]	(*p* = 0.052)
	F	158 [139.0; 159]		4 [3; 6]	

Data expressed as median [*q*1; *q*3]. Mann-Whitney Test; (*p*): *p* value.

**Table 5:** Correlation between nonverbal apraxia and verbal apraxia with age, education, and duration of PD.

	Total apraxia	
	Nonverbal apraxia	Verbal apraxia
	*r*_*s*_ (*p*)	*r*_*s*_ (*p*)
Age	0.425 (0.193)	0.169 (0.620)
Education	0.044 (0.898)	−0.619 (0.042)^**∗**^
Duration of PD	0.204 (0.547)	0.245 (0.468)

*r*_*s*_: Spearman's coefficient; (*p*): *p* value. ^**∗**^Correlation is significant if *p* value is less than 0.05.

## References

[1] Pals P., Van Everbroeck B., Grubben B. (2003). Case-control study of environmental risk factors for Parkinson's disease in Belgium. *European Journal of Epidemiology*.

[2] Armstrong R. A. (2011). Visual symptoms in Parkinson's disease. *Parkinson's Disease*.

[3] Ramig L. O., Countryman S., Thompson L. L., Horii Y. (1995). Comparison of two forms of intensive speech treatment for Parkinson disease. *Journal of Speech and Hearing Research*.

[4] Ramig L. O., Sapir S., Countryman S. (2001). Intensive voice treatment (LSVT) for patients with Parkinson's disease: a 2 year follow up. *Journal of Neurology Neurosurgery & Psychiatry*.

[5] Cardoso M. C. A. F., Goulart A. P. F., Marques D. F., Morisso M. F., Oliveira P. P. (2002). Xerostomia: sensação ou hipoprodução das glândulas salivares?. *Pró-Fono Revista de Atualização Científica*.

[6] Schindler J. S., Kelly J. H. (2002). Swallowing disorders in the elderly. *Laryngoscope*.

[7] Oliveira C. R., Ortiz K. Z., Vieira M. M. (2004). Disartria: estudo da velocidade de fala. *Pró-Fono Revista de Atualização Científica*.

[8] Vitorino M. R., Homem F. C. B. (2001). Doença de Parkinson: da fonação à articulação. *Fono Atual*.

[9] Darley F. L., Aronson A. E., Brown J. R. (1969). Clusters of deviant speech dimensions in the dysarthrias. *Journal of Speech and Hearing Research*.

[10] Fox C., Halpern A., Petska J., Spielman J., Will L., Ramig L. (2005). Voice treatment (LSVT) for individuals with Parkinson disease: new horizons. *Perspectives on Voice and Voice Disorders*.

[11] Spencer K. A., Rogers M. A. (2005). Speech motor programming in hypokinetic and ataxic dysarthria. *Brain and Language*.

[12] Shih L. C., Piel J., Warren A. (2012). Singing in groups for Parkinson's disease (SING-PD): a pilot study of group singing therapy for PD-related voice/speech disorders. *Parkinsonism and Related Disorders*.

[13] Pramstaller P. P., Marsden C. D. (1996). The basal ganglia and apraxia. *Brain*.

[14] Howard L. A., Binks M. G., Moore A. P., Playfer J. R. (2000). The contribution of apraxic speech to working memory deficits in Parkinson's disease. *Brain and Language*.

[15] Ortiz K. Z., Ortiz K. Z. (2010). Apraxia de fala. *Distúrbios Neurológicos Adquiridos: Fala e Deglutição*.

[16] Mateer C. (1978). Impairments of non-verbal oral movements after left hemisphere damage: a followup analysis of errors. *Brain and Language*.

[17] Darley F. L., Aronson A. E., Brown J. R., Darley F. L., Aronson A. E., Brown J. R. (1978). Apraxia para el habla: deficiencia en la programación motora del habla. *Alteraciones Motrices del Habla*.

[18] McNeil M. R., Robin D. A., Schmidt R. A., McNeil M. R. (2009). Apraxia of speech: definition, differentiation, and treatment. *Clinical Management of Sensorimotor Speech Disorders*.

[19] Hoehn M. M., Yahr M. D. (1967). Parkinsonism: onset, progression and mortality. *Neurology*.

[20] Martins F. C., Ortiz K. Z. (2004). Proposta de protocolo para avaliação da apraxia de fala. *Revista Fono Atual*.

[21] Fracassi A. S., Gatto A. R., Weber S., Spadotto A. A., Ribeiro P. W., Schelp A. O. (2011). Adaptação para a língua Portuguesa e aplicação de protocolo de avaliação das disartrias de origem central em pacientes com Doença de Parkinson. *Revista CEFAC*.

[22] Mac-Kay A. P. M. G., Mac-Kay A. P. M. G., Assêncio-Ferreira V. J., Ferri-Ferreira T. M. S. (2003). Dispraxia e disartria. *Afasias e demências: avaliação e tratamento fonoaudiológico*.

[23] Louis E. D., Wineld L., Fahn S., Ford B. (2001). Speech dysfluency exacerbated by levodopa in Parkinson’s disease. *Movement Disorders*.

[24] Shin H. Y., Lee W. Y., Suh M. K. (2007). Palilalia as a symptom of levodopa-induced oromotor hyperkinesia in Parkinson’s disease. *Parkinsonism and Related Disorders*.

[25] Benke T., Butterworth B. (2001). Palilalia and repetitive speech: two case studies. *Brain and Language*.

[26] Leiguarda R. C., Pramstaller P. P., Merello M., Starkstein S., Lees A. J., Marsden C. D. (1997). Apraxia in Parkinson's disease, progressive supranuclear palsy, multiple system atrophy and neuroleptic-induced parkinsonism. *Brain*.

[27] Ozsancak C., Auzou P., Dujardin K., Quinn N., Destée A. (2004). Orofacial apraxia in corticobasal degeneration, progressive supranuclear palsy, multiple system atrophy and Parkinson's disease. *Journal of Neurology*.

[28] Johns D. F., Darley F. L. (1970). Phonemic variability in apraxia of speech. *Journal of Speech and Hearing Research*.

[29] Wertz R. T., Lapointe L. L., Rosenbek J. C. (1984). *Apraxia of Speech in Adults: The Disorder and Its Management*.

[30] Cera M. L., Ortiz K. Z. (2009). Análise fonológica dos erros da apraxia adquirida de fala. *Pró-Fono Revista de Atualização Científica*.

[31] Darley F. L., Aronson A., Brown J. R. (1975). *Motor Speech Disorders*.

[32] Canter G. J., Trost J. E., Burns M. S. (1985). Contrasting speech patterns in apraxia of speech and phonemic paraphasia. *Brain and Language*.

[33] Metter E. J., Metter E. J. (1991). Relação cortical dos distúrbios da fala. *Distúrbios da Fala: Avaliação Clínica e Diagnóstico*.

[34] Brabo N. C., Minett T. S. C., Ortiz K. Z. (2014). Fluency in Parkinson's disease: disease duration, cognitive status and age. *Archives of Neuropsyquiatry*.

[35] Andrade C. R. F., Martins V. O. (2010). Variação da fluência da Fala em Idosos. *Pró-Fono*.

[36] Parente M. A. M. P., Scherer L. C., Zimmermann N., Fonseca R. P. (2009). Evidências do papel da escolaridade na organização cerebral. *Revista Neuropsicologia Latinoamericana*.

[37] Da Silva S. L. (2004). Reabilitação Neuropsicológica em Idosos, ‘Uma gota no oceano’. *Revista Eletrônica Com Ciência*.

[38] Da Silva S. L., Coelho D. S., Alchieri J. C., Landeira-Fernadez J., Silva M. T. A. (2007). Plasticidade cerebral, meio ambiente e comportamento e cognição: bases aliadas às neurociências para o estudo da reabilitação neuropsicológica da memória. Intersecções entre Psicologia e Neurociências. *Intersecções entre Neurociência e Psicologia*.

[39] Dronkers N. F. (1996). A new brain region for coordinating speech articulation. *Nature*.

[40] Maciel Júnior J. A., Capovilla F. C., Gonçalves M. J., Macedo E. C. (1998). Reabilitação das Apraxias. *Tecnologia em (re) habilitação cognitiva: uma perspectiva multidisciplinar*.

[41] Della Sala S., Basso A., Laiacona M., Papagno C., Vallar G., Cappa S. F., Wallesch C. W. (1992). Subcortical localization of ideomotor apraxia: a review and an experimental study. *Neuropsychological Disorders Associated with Subcortical Lesions*.

[42] Boller F., Mizutani T., Roessmann U., Gambetti P. (1980). Parkinson disease, dementia, and Alzheimer disease: clinicopathological correlations. *Annals of Neurology*.

[43] Hughes A. J., Daniel S. E., Kilford L., Lees A. J. (1992). Accuracy of clinical diagnosis of idiopathic Parkinson's disease: a clinico-pathological study of 100 cases. *Journal of Neurology, Neurosurgery & Psychiatry*.

[44] Hughes A. J., Daniel S. E., Blankson S., Lees A. J. (1993). A clinicopathologic study of 100 cases of Parkinson's disease. *Archives of Neurology*.

[45] Uyama N., Yokochi F., Bandoh M., Mizutani T. (2013). Primary progressive apraxia of speech (AOS) in a patient with Pick's disease with Pick bodies: a neuropsychological and anatomical study and review of literatures. *Neurocase*.

[46] Lobo P. P., Pinto S., Rocha L., Reimão S., de Carvalho M. (2013). Orofacial apraxia in motor neuron disease. *Case Reports in Neurology*.

[47] Rohrer J. D., Rossor M. N., Warren J. D. (2010). Apraxia in progressive nonfluent aphasia. *Journal of Neurology*.

[48] Luria A. R. (1983). *Las funciones corticales del hombre: alteraciones de las funciones corticales superiores por lesion cerebral*.

[49] Vargha-Khadem F., Watkins K. E., Price C. J. (1998). Neural basis of an inherited speech and language disorder. *Proceedings of the National Academy of Sciences of the United States of America*.

[50] Ogar J., Slama H., Dronkers N., Amici S., Gorno-Tempini M. L. (2005). Apraxia of speech: an overview. *Neurocase*.

[51] Dias A. E., Chien H. F., Barbosa E. R. (2011). O método Lee Silverman para reabilitação da fala na doença de Parkinson. *Revista Neurociências*.

[52] Kandel E. R., Schwartz J. H., Jessell T. M. (2003). *Princípios da neurociência*.

[53] Teive H. A. G., Meneses M. S., Meneses M. S., Teive H. A. G. (1996). Histórico. *Doença de Parkinson: aspectos clínicos e cirúrgicos*.

[54] Frank C., Pari G., Rossiter J. P. (2006). Approach to diagnosis of Parkinson disease. *Canadian Family Physician*.

